# Professional altruism in nursing care: A concept clarification study

**DOI:** 10.1016/j.ijnsa.2026.100522

**Published:** 2026-03-16

**Authors:** Anna Slettmyr, Anna Schandl, Maria Arman, Karin Hugelius

**Affiliations:** a School of Health Sciences, Faculty of Medicine and Health, Örebro University, Fakultetsgatan 1, 701 82 Örebro, Sweden; b Department of Neurobiology, Care Sciences and Society, Karolinska Institutet, Alfred Nobels Allè 23, 141 52 Huddinge, Sweden; c Department of Clinical Science and Education, Södersjukhuset, Karolinska Institutet, Sjukhusbacken 10, 118 83 Stockholm, Sweden; d Department of perioperative Care and Intensive Care, Södersjukhuset, Sjukhusbacken 10, 118 83 Stockholm, Sweden

**Keywords:** Altruism, Concept clarification, Ethics, Nursing care, Nursing values

## Abstract

**Background:**

Altruism has historically shaped the ethos of nursing. However, the COVID-19 pandemic reignited nurses’ profound commitment to patient care, often at significant personal risk to own health and safety. This renewed dedication has prompted interest in whether altruism remains a vital component of nursing practice.

**Objective:**

To clarify the meaning of professional altruism in nursing care.

**Design:**

Catherine Norris’s five-step concept clarification method was employed.

**Methods:**

A systematic search was conducted in November 2024 across the CINAHL, PubMed, MEDLINE, and PsycINFO databases using the terms *altruism, altruistic, and altruistic behavior/behaviour* in combination with *nurses, nursing*, and *nursing care*, resulting in the inclusion of 24 articles.

**Results:**

Systemised descriptions of professional altruism yielded five categories: a *willingness to act for others, a moral orientation, a motivational force, an unwavering professional expectation*, and *a valued, yet challenged and sometimes rejected phenomenon*. Additionally, an operational definition of professional altruism in nursing care emerged: *Professional altruism is a moral orientation toward fellow human beings in need of care, characterised by a willingness to prioritise the well-being of others over one’s own needs. While balancing the expectations, challenges, and personal consequences involved, professional altruism remains a core aspect of nursing care, responsibility, and practice*.

**Conclusion:**

Professional altruism is a central aspect of nurses’ professional identity and an essential element of nursing care. When acknowledged and supported, professional altruism can enhance both the quality of care and nurses’ well-being.

**What is already known**

 

• Historically, altruism has shaped nursing ethos, with compassion and responsibility driving nurses to act selflessly for others.
• Over time, natural caring has been replaced by professionalised care.
• The COVID-19 pandemic brought renewed attention to nurses’ dedication to and motivation for providing care.

**What this paper adds**

 

• We have shown that altruism remains an important aspect of nurses’ professional identity and nursing care.
• Professional altruism is challenged within care delivery contexts and poses risks to nurses’ health and well-being.
• In addition, we have proposed an operational definition of professional altruism and presented a model to support further empirical research.

Alt-text: Unlabelled box dummy alt text

## 1 Background

This concept clarification focuses on altruism, which is generally understood as a universal moral phenomenon defined by selfless concern for fellow humans and the prioritisation of others’ needs over one’s own. Within nursing care, altruism is recognised not only as a moral ideal but also as a core professional value, mediated through nursing education and professional culture (Milton, 2012). Historically, altruism has shaped nursing ethos, where compassion and responsibility have driven nurses to act selflessly for others (Kuhse et al., 1999; Sarvimäki and Stenbock-Hult, 2008). According to the International Council of Nurses (2021), nursing care, as a humanistic scientific discipline, involves a commitment to caring for individuals with the aim “to promote health, to prevent illness, to restore health, and to alleviate suffering and promote a dignified death” (p.2). The nursing profession is grounded in a theoretical framework in which concepts play a crucial role in affirming, understanding, and acknowledging the various phenomena that arise within the nurse–patient encounter and the caring relationship (Meleis, 2018). Caring is recognised as a universal and natural phenomenon in nursing, with human caring serving as an ontological foundation for shared humanity (Eriksson, 2018). The philosophy of caring science conveys the values and beliefs fundamental to all caregiving disciplines, providing a framework and worldview through which both ontological and epistemological questions can be explored. These questions pertain to the core values, assumptions, concepts, propositions, and actions that define and guide the discipline of nursing care (Meleis, 2018). Historically, the altruistic dimension of nursing care has also been closely associated with the ideals of motherhood and femininity. This association has contributed to the exploitation of nurses by framing nursing care as an extension of personal traits, such as kindness, patience, and unconditional love, that are expected to be demonstrated selflessly by nurses (White, 2002). With the evolution of society, natural or instinctive caring has increasingly been replaced by professionalised forms of care (Eriksson, 2018). Professional nursing care represents an ethical commitment grounded in experience and knowledge, emphasising attentiveness and a willingness to act in the patient’s best interest. Central to this commitment is unselfish engagement, in which the caregiver prioritises patient needs (Martinsen, 2000). Contemporary healthcare in the Western societies is increasingly driven by financial incentives, efficiency demands, streamlined workflows, and the imperative to maintain balanced budgets. These conditions may force nurses to distance themselves from patients, limiting engagement and authentic encounters and thereby constraining the opportunities for professional altruism (Martinsen, 2006). In addition, the nursing profession is increasingly perceived as salaried labour, reflecting a shift from its altruistic roots (Straughair, 2012). Although the COVID-19 pandemic reignited nurses’ profound commitment to patient care (Slettmyr et al., 2023; Tingsvik et al., 2024; Tong et al., 2021), this dedication often came at significant personal risk and generated internal conflict as nurses balanced their moral and professional duty to care against threats to their own health, safety, and long-term well-being (Slettmyr et al., 2025; Tingsvik et al., 2024). This reported ambivalence toward professional altruism has renewed scientific interest in the phenomenon, prompting reflection on whether it is a fading ideal of the past or remains a vital component of contemporary healthcare practice.

### 1.1 Objective

We aimed to clarify the meaning of professional altruism in nursing care.

## 2 Methods

### 2.1 Design

We employed the concept clarification method, which aims to refine concepts and strengthen conceptual frameworks (Norris, 1982). Concept clarification explores the internal relationships within a concept, identifies new relationships, and resolves ambiguities (Meleis, 2018). By articulating an operational definition and developing a model suitable for empirical research, this method broadens the understanding of a phenomenon from a clinical perspective (Meleis, 2018). We focused on clarifying the meaning of professional altruism in nursing care, particularly how nurses experience it in patient encounters. The method was chosen for its close alignment with clinical practice (Meleis, 2018). Grounded in empirical data, concept clarification treats the concept as a symbolic representation and ultimately informs the management of the phenomenon in clinical practice (Lackey, 2000). We specifically examined professional altruism within the context of nursing care and did not address personal altruistic actions outside the profession.

### 2.2 Data collection

A systematic literature search was conducted by the first author in collaboration with an academic librarian across the CINAHL, PubMed, MEDLINE, and PsycINFO databases. The search used the terms *altruism, altruistic, and altruistic behavior/behaviour*, in combination with *nurses, nursing*, and *nursing care,* using the Boolean operators AND and OR. Limits were applied to include only peer-reviewed articles published in English. The database searches were conducted on November 13, 25, and 26, 2024. For a detailed description of the search strategy and results, see Supplementary Material File 1.

The inclusion criteria were as follows: 1) original qualitative, quantitative, or mixed-method studies exploring the meaning or expressions of altruism in clinical contexts and in nurse-patient caring encounters, 2) peer-reviewed publications, and 3) articles published in English. Exclusion criteria were editorials, theoretical papers addressing the clinical aspects of altruism, and articles based solely on personal reflections.

### 2.3 Data selection

The initial database search yielded 3045 articles. All references were imported into Covidence systematic review software, which automatically identified and removed duplicates. This resulted in 1435 unique articles that were screened by title and abstract by the first author (ASl) to select articles suitable for the concept clarification. Thereafter, the first and last authors (ASl and KH) conducted a full-text screening of 60 articles. Any disagreements were resolved through discussion. This process resulted in 24 articles being included in the concept clarification.

**Fig. 1:**
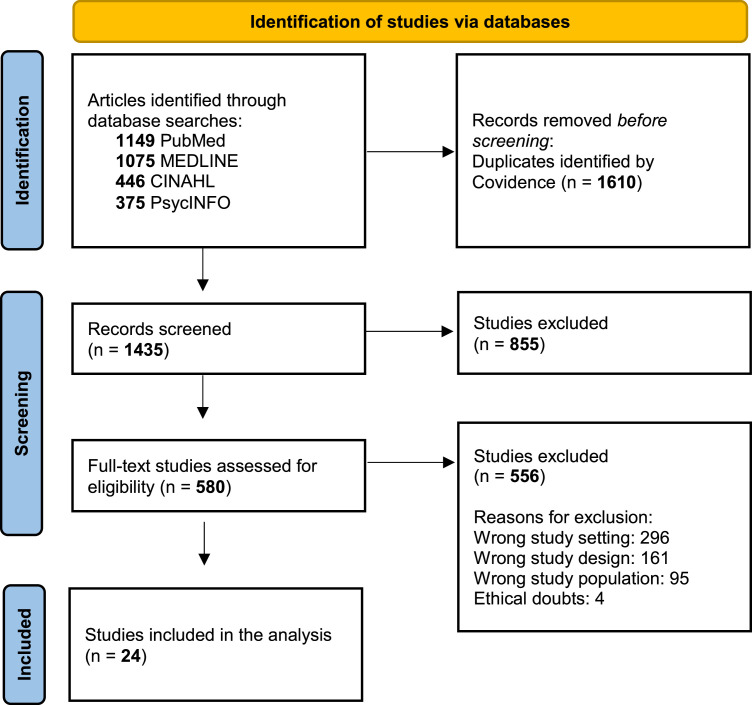
PRISMA Flowchart – the data selection process.

The data selection process is illustrated in Fig. 1, and a matrix of the included articles is presented in Supplementary Material File 2.

### 2.4 Analysis

For analysis, we followed the five-step process outlined in the concept clarification method (Norris, 1982). In the first step, we identified, observed, and described the phenomenon of professional altruism using the included articles, previously conducted concept analyses, and dictionary definitions. In the second step, a systematic analysis of the data rendered from the 24 articles was conducted. Relevant data were extracted and inductively organised into categories and subcategories, with consideration of their internal hierarchies. The first author (ASl) performed this step and repeatedly discussed and validated it with the other authors. In the third step, all authors collaboratively formulated an operational definition of professional altruism, guided by the question ‘How will I know the concept when I see it?’ (Norris, 1982). In the fourth step, a conceptual model was developed to illustrate the relationships among the identified categories. Finally, the fifth step involved formulating hypotheses to clarify the concept in relation to its application in future empirical research (Meleis, 2018; Norris, 1982). Throughout the analysis, any disagreements were resolved through discussion to reach consensus within the research team.

## 3 Results

In all, 24 original research articles were included in this concept clarification study. Of these, 17 used a qualitative study design, six employed a quantitative study design, and one utilised a mixed-methods approach. The studies were conducted across multiple countries, including Iran (*n* = 7), Sweden (*n* = 3), the United States of America (*n* = 3), Turkey (*n* = 2), and one study each from Australia, Belgium, China, Germany, Indonesia, Israel, Kuwait, Norway, and the United Kingdom. Publication dates spanned from 1997 to 2025. The studies were carried out in various clinical contexts, including paediatric care (*n* = 3), COVID-19 care (*n* = 3), acute care settings (*n* = 2), oncology (*n* = 2), and one study each in community nursing, humanitarian work, long-term care, mental health, orthopaedic wards, and transitional care. Eight studies did not specify a particular setting (*n* = 8). For detailed characteristics of all included articles, see Supplementary Material File 2.

### 3.1 Step one: observing and describing the phenomenon

The term altruism originates from the French word *autrui* (‘somebody else’ or ‘others’), which is derived from the Latin *alter* (‘other) (Oxford Dictionary, 2025). The Oxford Dictionary (2025) defines altruism as ‘*disinterested or selfless concern for the well-being of others, especially as a principle of action*’ with antonyms including ‘*selfishness, egoism, or (in early use) egotism’*. The Cambridge Dictionary (2025) offers two definitions: ‘*willingness to do things that bring advantages to others, even if it results in disadvantages for yourself*’, and ‘*the attitude of caring about others and doing acts that help them although you do not get anything by doing those acts’*. In both dictionaries, altruism is contrasted with egoism and self-interest.

In social science, altruism is not considered a uniquely human trait but rather a biological phenomenon observed in animals and nature, reflecting a broad and naturalistic perspective. It can be interpreted through both individual and collective lenses, understood not merely as a behaviour or motivation but also as a moral and social norm embedded within society (Bykov, 2017).

In biology, the concept of reciprocal altruism refers to situations in which individuals remember past interactions, and the cost to the helper is outweighed by the benefit to the recipient. Altruism has also been linked to natural selection, where actions that enhance the survival and reproductive success of genetically-related individuals are favoured (APA Dictionary of Psychology, 2025; Dowd et al., 2007).

In psychology, altruism encompasses a wide range of behaviours intended to benefit others at a personal cost, including actions such as volunteerism and martyrdom (APA Dictionary of Psychology, 2025). Altruism can also be understood as a culturally-shaped behaviour emerging from shared values and beliefs within a group, cultivated through socialisation and transmitted both within and across generations (Dowd et al., 2007).

In a previous analysis of the concept of altruism within caring, several critical attributes were identified (Smith, 1995). These included a sense of responsibility for the well-being of others, compassion for fellow human beings, empathy, the ability to adopt another’s perspective, and an uncalculated selfless commitment to prioritising others’ needs over one’s own. The consequences of altruistic behaviour included feelings of satisfaction derived from others’ happiness or well-being, a sense of relief when others’ needs are met, and the perception that caring for others is inherently valuable (Smith, 1995).

Altruism has been conceptualised as a core personal value in nursing by many researchers and is described as a value rooted in concern for the welfare of others, fostering a willingness to serve regardless of potential personal consequences (van der Wath and van Wyk, 2020). A contemporary definition of altruism in nursing emphasises helping others without expectation of reward, prioritising the patient’s interests and striving to meet ethical obligations while balancing self-sacrifices with personal well-being (Chen et al., 2022). Arman (2023) characterised altruism as ‘an expression of will to act for the good’, metaphorically described as ‘the hands’ that carry out caring actions. Furthermore, altruism has been linked to the concept of agape, a form of selfless love for humanity, which underpins the ethical imperative in nursing to care for strangers as if they were loved ones (Dowling, 2004).

Within clinical nursing care, altruism is frequently described as a professional responsibility (Albuquerque et al., 2018; Hamooleh et al., 2013), reflecting a willingness to prioritise the needs of fellow human beings over one’s own (Alavi et al., 2015; Decoyna et al., 2018; Fagermoen, 1997; Pang et al., 2009; Slettmyr et al., 2022, 2019). It is characterised by an other-oriented attitude (Fagermoen, 1997), a moral orientation grounded in personal values, and a desire to help fellow human beings (Cross et al., 2020; Fagermoen, 1997; Slettmyr et al., 2022; Vishnevsky et al., 2015). This orientation often emerges through encounters with, and identification with, others (Decoyna et al., 2018; Fagermoen, 1997; Hamooleh et al., 2013; Shahoei et al., 2022; Slettmyr et al., 2019; Zarea et al., 2013) and may be inspired or reinforced by religious beliefs (Atkinson, 2015; Rantung, 2025; Zarea et al., 2013). Altruism in nursing involves personal sacrifices (Alavi et al., 2017; Decoyna et al., 2018; Pang et al., 2009; Shahoei et al., 2022; Slettmyr et al., 2022), and selfless actions (Vishnevsky et al., 2015) that exceed formal duties, often undertaken voluntarily or by doing the little extra for others (Ghaljeh et al., 2024; Ghanbari-Afra et al., 2021; Slettmyr et al., 2019). It is described as both a habitual and natural aspect of the nursing profession (Altun, 2002; Atkinson, 2015; Carter, 2014; Lazar, 2010; Slettmyr et al., 2019) and a societal expectation placed upon nurses (Slettmyr et al., 2019; Özsaban et al., 2024).

In addition, altruism is considered an integral element of the caring relationship (Alavi et al., 2015; Atkinson, 2015; Decoyna et al., 2018; Ghaljeh et al., 2024; Ghanbari-Afra et al., 2021; Hamooleh et al., 2013; Vishnevsky et al., 2015). It serves as a motivator for entering and remaining in the nursing profession (Alavi et al., 2015; Albuquerque et al., 2018; De Cooman et al., 2008; Rantung, 2025; Shahoei et al., 2022; Slettmyr et al., 2019; Zarea et al., 2013; Özsaban et al., 2024) and contributes to a sense of meaning and job satisfaction (Carter, 2014; Cross et al., 2020; Dotson et al., 2014; Eder and Meyer, 2023; Lazar, 2010; Slettmyr et al., 2019). Altruism is frequently discussed alongside related concepts, such as commitment (Altun, 2002; Atkinson, 2015; Özsaban et al., 2024), sympathy (Altun, 2002; Shahoei et al., 2022), empathy (Decoyna et al., 2018; Ghaljeh et al., 2024; Ghanbari-Afra et al., 2021; Hamooleh et al., 2013; Slettmyr et al., 2019) generosity (Altun, 2002), benevolence (Altun, 2002; Vishnevsky et al., 2015), humaneness (Fagermoen, 1997), and love for humanity (Zarea et al., 2013). It is also described as a key component of family-centred care (Alavi et al., 2015; Ghaljeh et al., 2024) and ethics-based care (Hamooleh et al., 2013).

### 3.2 Step two: systemising observations and descriptions

The systematic analysis of the data yielded five categories with associated subcategories, as presented in Table 1.

**Table 1:** Professional altruism in nursing care: categories and sub-categories.

Categories	*A willingness to act for others*	*A moral orientation*	*A motivational force*	*An unwavering professional expectation*	*A valued, yet challenged and sometimes rejected phenomenon*
**Sub-categories**	Prioritising the needs of others over one’s own	Arises from personal values and occasionally inspired by religious beliefs	Contributes to job satisfaction	A societal expectation placed on the nursing profession	Hampered by organisational demands
	Making sacrifices inherent to the nursing profession	An inner driving force for caring	Generates valued rewards	A fundamental value of nursing	Challenged by personal beliefs about the nursing profession
	‘Doing the little extra’	Identifying with ‘the other’	Provides positive outcomes for nursing	An important and prioritised professional value	May compromise the health and well-being of nurses

An example of the systematic analysis is provided in Supplementary Material File 3. An overview of references represented across the different categories in step two is provided in Supplementary Material File 4.

#### 3.2.1 A willingness to act for others

Professional altruism in nursing care was expressed as a willingness to prioritise the needs, safety, health, and life of others over one’s own, often going beyond ordinary measures to benefit someone else.

Professional altruism meant *prioritising the needs of others over one’s own*, shifting focus from self to others, demonstrating selflessness, and making a personal effort to meet the needs of fellow human beings. Including taking responsibility and advocating for them. This orientation was characterised by a willingness to care for others with a positive compassionate approach.

Professional altruism also implied *making sacrifices inherent to the nursing profession*, such as compromising personal safety or interests, and was often described as a natural aspect of nursing. However, nurses noted the challenge of determining boundaries for reckless or extreme self-sacrifices within professional practice. Altruistic actions and sacrifices varied in scale and impact, affecting patients, their families, and the nurses themselves.

Professional altruism was also described as “*doing the little extra”*’, reflecting actions that exceed one’s professional duties without being explicitly requested.

#### 3.2.2 A moral orientation

As a moral orientation, professional altruism was associated with feelings of compassion, sympathy, and empathy for others, rooted in personal values and, at times, influenced by religious beliefs.

Professional altruism *arises from personal values and occasionally inspired by religious beliefs* and was described as aligned with one’s moral compass—an overarching value guiding nursing practice. It was also viewed as a personal trait, reflecting a genuine desire to help others and derive satisfaction from doing so. From this perspective, altruism encompasses a holistic view of humanity, characterised by humaneness, presence, understanding, attentive listening, and the provision of support and guidance. In certain cultural contexts, an altruistic approach to caring was deeply rooted in religious beliefs, motivated by love for humanity and the belief that God would reward such compassion.

Professional altruism functioned as *an inner driving force for caring*, a powerful driver for self-efficacious nurses, and an intrinsic value underpinning professional commitment to nursing practice. It also guided care during crises, such as pandemics, reinforcing nurses’ dedication to patients.

Additionally, professional altruism was described as *identifying with ‘the other’*, patients as well as their families, shaped by close professional engagement and the emotional demands of caregiving. This identification, often emerging from direct clinical interactions, was influenced by compassion, sympathy, and empathy. When nurses connected personally with those in their care, altruism became both a moral orientation and an emotional response rooted in shared humanity.

#### 3.2.3 A motivational force

Professional altruism contributed to a sense of meaningfulness at work. It fostered feelings of making a difference in others’ lives acting out of benevolence, which often led to a sense of personal achievement.

Professional altruism *contributes to job satisfaction,* and nurses who were more altruistic reported greater work satisfaction.

Altruistic behaviour *generates valued rewards*, including fulfilment, joy, liveliness, and a sense of spiritual connection, sometimes experienced as a closer relationship with God or similar power. It also enhanced the meaningfulness of work and reinforced the perception of making a difference for patients in caring encounters. Acting altruistically and receiving criticism occasionally strengthened the feeling of altruism and the willingness to make sacrifices for others. At the same time, nurses expressed ambivalence between selflessness/self-sacrifice and deriving satisfaction from positive outcomes when helping others. Professional altruism was also associated with personal growth and accomplishment, as benevolent actions toward others reinforced both self-oriented intellectual stimulation and other-oriented values, such as humaneness and attentiveness to patients’ needs.

Professional altruism *provides positive outcomes for nursing.* An altruistic approach enhanced nursing care quality, patient safety, and patient satisfaction. It played a crucial role in crises, such as during the COVID-19 pandemic, when nurses prioritised patients’ needs over their own health. Altruistic commitment toward patients and their families also proved to be an important driving force for the sustainability of nursing practice in healthcare, both during routine care and in crises situations.

#### 3.2.4 An unwavering professional expectation

Professional altruism was perceived as a core professional value in nursing care, deeply embedded in both societal and professional expectations and integral to nursing culture.

Professional altruism reflects *a societal expectation placed on the nursing profession*, where caring for patients, especially in times of crisis such as a pandemic, is often seen as a self-evident responsibility. This expectation comes not only from society at large but also from patients and their families, reinforcing the perception of nurses as inherently-benevolent caregivers.

Professional altruism was expressed as *a fundamental value of nursing,* central to nursing culture, clinical practice, the nurse–patient relationship, and the provision of benevolent, ethic-based care. It encompasses taking responsibility, accepting patients and their unique needs, and demonstrating trustful, supportive behaviour toward others.

Altruism was further recognised as *an important and prioritised professional value*, forming part of the broader value system in nursing care and serving as a professional quality that reinforces nurses’ ethical foundation. Professional altruism also shaped nurses’ professional lives: it was a natural aspect of their work, grounded in knowledge, experience, and compassion for others. Moreover, it represented a fundamental humanistic element: encompassing pro-social behaviour and constituting an essential component of providing compassionate care.

#### 3.2.5 A valued, yet challenged and sometimes rejected phenomenon

Professional altruism was often challenged by not only organisational and economic pressures but also the personal sacrifices and high internal demands placed on nurses, often resulting in emotional strain and personal suffering.

In daily practice, professional altruism was *hampered by organisational demands*, such as regulations, workload demands, stressful environments, and insufficient staffing. Nurses sometimes considered leaving the profession when unable to fulfil their altruistic intentions, highlighting the strong impact of altruism on their working experience.

Professional altruism was also *challenged by personal beliefs about the nursing profession.* While altruism was widely recognised as essential to nursing care, it was sometimes personally rejected when it conflicted with the perception of nursing as salaried work rather than a calling. As a result, altruism could simultaneously be seen as a prerequisite for professional practice and as a source of tension or uncertainty.

Furthermore, professional altruism *may compromise the health and well-being of nurses,* increasing the risk of self-endangering behaviours, exhaustion, or compassion fatigue. Challenges included setting boundaries and managing obligations that extended beyond professional duties. These factors were associated with higher intentions among nurses to leave the profession.

### 3.3 Step three: obtaining a definition

Professional altruism is a broad phenomenon grounded in nurses’ morals and values, emerging in encounters with patients and their families. Confronting patients’ vulnerability and suffering, as well as managing expectations and challenges, fosters a willingness to prioritise their needs, make sacrifices, and go beyond expected duties.

Based on the analysis, the following operational definition is proposed:

*Professional altruism is a moral orientation toward fellow human beings in need of care, characterised by a willingness to prioritise the well-being of others over one’s own needs. While balancing the expectations, challenges, and personal consequences involved, professional altruism remains a core aspect of nursing care, responsibility, and practice.*

### 3.4 Step four: producing a model

**Fig. 2:**
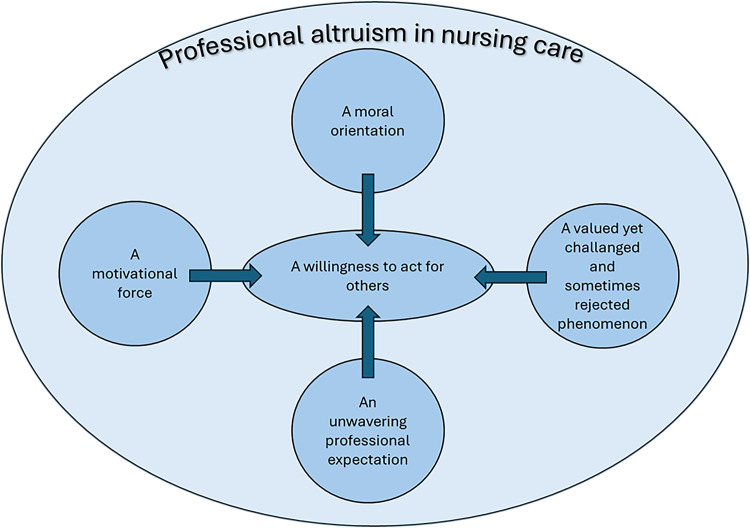
Professional altruism in nursing care - a model.

A model of the components of professional altruism is presented in Fig. 2. It depicts the relationships among the categories described in the second step and in the proposed definition. At the core of the model is the willingness to act for others, which derives actual altruistic actions that support the well-being of patients and their families. This willingness is shaped by the nurse’s moral orientation, perceived consequences of altruistic actions, professional expectations, and the challenges encountered when applying altruism in clinical nursing care.

The willingness to act both inspires and strengthens moral responsibility. When nurses engage willingly in nursing care, it validates their ethical stance, and intrinsic motivators further strengthen their commitment. Professional altruism is a fundamental component of nurses’ professional identity and values, functioning as a moral orientation within the nurse-patient relationship. Nurses often feel ethically-compelled to act in the best interests of their patients reinforcing altruistic behaviour as not merely a personal choice but a moral necessity. The professional culture of nursing care also establishes altruism as an expected norm, shaping identity and behaviour and reinforcing it as a core professional value. Through education and clinical experience, professional altruism becomes a natural, habitual response that sustains nurses’ purpose and commitment to care. However, contextual challenges can hinder enactment of altruistic actions, highlighting the tension between moral intent and practical feasibility.

### 3.5 Step five: formulating hypotheses

Based on the concept clarification, the following hypotheses about professional altruism are proposed for further study:

• In a clinical context, professional altruism may emerge spontaneously in response to patients’ vulnerability, needs, and suffering.
• Altruism remains a significant component of nurses’ professional identities, nursing culture, and the practice of nursing care.
• Altruistic behaviour may enhance the quality of care, including ethically-grounded care, patient safety, and patient satisfaction.
• Supporting nurses’ altruistic impulses and actions can enhance job satisfaction, foster a sense of meaning in their work, contribute to their well-being, and improve retention within the profession.
• Organisational factors, including leadership, cultural norms, and structural conditions, play important roles in either facilitating or hindering the expression of professional altruism in health care settings.

## 4 Discussion

From this clarification of the concept of professional altruism, we suggest that altruism remains an important and inherent aspect of nursing care, aligning with previous studies on the phenomenon (Chen et al., 2022; Smith, 1995; van der Wath and van Wyk, 2020). It reflects the foundational ethical dimension of nursing and the courage to engage in authentic human encounters (Martinsen, 2000). Understanding and modelling the current meaning and clinical relevance of professional altruism can guide future research on nurses’ willingness to care both in everyday nursing practice and crises situations. Although the duty to care is legally mandated for nurses, the ethical dimension of nursing arises from a spontaneous response to human vulnerability and suffering (Martinsen, 2012). Fostering and supporting nurses’ altruistic intentions could help build cultural norms that enhance job satisfaction, provide meaning, and impact patients positively (Martinsen, 2012). Yu et al. (2025) reported in a systematic review that resilience among nurses during the COVID-19 pandemic was linked to perceptions of a supportive organisational culture that promoted compassion, engagement, and caring behaviour. We have highlighted professional altruism as a valuable element of nursing care, benefitting not only nurses but also patients, health care organisations, and society—by improving care quality, patient satisfaction, and safety. Conversely, failing to support nurses in their altruistic efforts may result in moral stress, compassion fatigue, and decreased resilience (Yu et al., 2025). Such negative outcomes for nurses may also adversely affect patient outcomes and long-term organisational sustainability, including increased nurse turnover (Boulton et al., 2025; Brown et al., 2024).

Already 20 years ago, Martinsen (2006) highlighted the importance of reflecting on which values, norms, and phenomena should guide and strengthen the nursing knowledge base, culture, and clinical practice in a changing health care system. Today, nurses continue to face similar challenges as patients’ needs intersect with organisational demands, including standardised protocols (Van Mersbergen-de Bruin et al., 2025). Prevailing norms in health care may either support or hinder nurses’ capacity for spontaneous altruistic actions and their confidence in responding to ethical demands that arise in encounters with vulnerable individuals (Martinsen, 2000). In contrast, when nurses’ altruistic instincts and willingness are hindered or unsupported, patients risk being viewed without recognition of their individuality and suffering, and nursing care may unintentionally contribute to further suffering (Martinsen, 2006). While a concept may become ‘outdated’, the underlying phenomenon—in this case, professional altruism—can remain highly-relevant in clinical nursing practice. Updating definitions and understandings of such a phenomenon can influence nurses’ self-image and perceptions of their profession (Parastesh et al., 2025). Although nursing has evolved into an academic discipline grounded in evidence-based care, the traditional notion of professional altruism may no longer resonate with contemporary practitioners. Without clarification, this could diminish the perceived value of the profession. However, redefining professional altruism to align with modern nursing practice may reinforce its significance particularly in the context of current nurse shortages. Understanding what motivates nurses to enter, remain, and find meaning in the profession is crucial for the future sustainability of healthcare.

While professional altruism remains a natural and essential aspect of nursing care, its role in contemporary healthcare warrants further reflection. As both a personal and professional value embedded in individual nurses and the broader nursing culture, professional altruism should be actively supported to prevent negative consequences, such as emotional exhaustion or compassion fatigue. Strengthening support for professional altruism can also enhance workforce retention by safeguarding nurses’ well-being and enabling sustainable care practices (Jiang et al., 2022). Furthermore, altruistic care not only benefits patients and families but also contributes positively to healthcare organisations and society at large. When effectively supported within nursing culture, professional altruism can create a ‘win-win’ scenario enhancing ethical, personalised, safe, and high-quality care while fostering nurses’ well-being, professional fulfilment and job satisfaction.

### 4.1 Strengths and limitations

A major strength of this concept clarification study was the systematic and comprehensive search strategy, which encompassed four major databases and yielded a substantial pool of potentially-relevant articles. This broad approach increased the likelihood that the analysis captured the full breadth of existing knowledge related to the concept. Another strength was the collaborative validation process (Flanagan et al., 2025). The research team jointly reviewed the study selection, coding decisions, analytical interpretations, and the development of definitions and model components. This collaborative approach enhanced consistency, reduced individual bias, and contributed to the credibility and coherence of the final conceptual model.

Despite this rigorous approach, several limitations should be noted. Relevant studies may have been missed due to indexing limitations, inconsistent terminology, or publication in sources not covered by the selected databases. The study did not include a formal appraisal of methodological quality, and the evidence base consisted largely of qualitative data focusing on nurses’ experiences, which may limit the conceptual range represented in the analysis. As with all secondary analyses relying on qualitative primary studies, the findings may have been influenced, directly or indirectly, by the interpretive perspectives of the original research teams (Tatano Beck, 2023). This risk was partially mitigated through the research team’s collective validation procedures. Additionally, the conceptual model developed in the fourth step has not yet undergone external review. Without external expert assessment or empirical testing, its applicability and credibility remain preliminary. Finally, some proposed conceptual linkages—particularly those connecting professional altruism with emotional exhaustion, fatigue, and quality of care—require further empirical investigation.

## 5 Conclusion

In this concept clarification study, we have shown that professional altruism is a central aspect of nurses’ professional identity, encompassing a moral commitment and a willingness to prioritise patients. We have highlighted that professional altruism remains a natural and important part of nursing care. When professional altruism is acknowledged and supported within the organisational environments, it can enhance both the quality of care and nurses’ sense of meaning and well-being. Conversely, insufficient organisational support may increase nurses’ vulnerability to exhaustion and compassion fatigue. Future researchers should explore what motivates nurses to enter the profession, remain in it, and find meaning throughout their careers, in order to better support and retain the nursing workforce. A supportive work environment, ethical organisational cultures, and educational opportunities for structured reflection on moral motivation may help sustain professional altruism as a positive and protective force in modern health care.

## Funding

This research did not receive any specific grant from funding agencies in the public, commercial, or not-for-profit sectors.

## CRediT authorship contribution statement

**Anna Slettmyr:** Writing – original draft, Visualization, Validation, Methodology, Formal analysis, Conceptualization. **Anna Schandl:** Writing – original draft, Visualization, Validation, Methodology, Formal analysis, Conceptualization. **Maria Arman:** Writing – original draft, Visualization, Validation, Methodology, Conceptualization. **Karin Hugelius:** Writing – original draft, Visualization, Validation, Methodology, Formal analysis, Conceptualization.

## Declaration of competing interest

Nothing to declare.

## Appendix Supplementary materials

Image, application 1

Image, application 2

Image, application 3

Image, application 4
