# Supplementary material for: Professional altruism in nursing care: A concept clarification study
Source: Int J Nurs Stud Adv. 2026 Mar 16;10:100522. doi: 10.1016/j.ijnsa.2026.100522 (PMC13053996; doi:10.1016/j.ijnsa.2026.100522)
Supplement: Supplementary file 1 [file mmc1.docx]

| **Supplementary Material File 1. Systematic search** | | | |
| --- | --- | --- | --- |
| **Database Searchterms** | | | **n** |
| **CINAHL Full text** 2024-11-13 | | | |
|  | 1. | (altruis*) | 4309 |
|  | 2. | (“altruistic behaviour*”) | 33 |
|  | 3. | (”altruistic behavior”) | 72 |
|  | 4. | (MH "Altruism") | 2870 |
|  | 5. | S1 OR S2 OR S3 OR S4 | 4302 |
|  | 6. | (Nurs*) | 957041 |
|  | 7. | (MH “Nurses) | 71075 |
|  | 8. | (“Nursing care”) | 68945 |
|  | 9. | (MH “Nursing care”) | 28127 |
|  | 10. | S6 OR S7 OR S8 OR S9 | 957041 |
|  | 11. | S3 AND S9 | 607 |
|  | 12. | Peer Reviewed; English Language | 445 |
|  |  |  |  |
| Medline 2024-11-25 | | |  |
| \|  \| 1. \| (altruis*) \| 13224 \| \| \| --- \| --- \| --- \| --- \| --- \| \|  \| 2. \| (“altruistic behaviour*”) \| 170 \| \| \|  \| 3. \| (”altruistic behavior*”) \| 512 \| \| \|  \| 4. \| (MH "Altruism") \| 8135 \| \| \|  \| 5. \| S1 OR S2 OR S3 OR S4 \| 13224 \| \| \|  \| 6. \| (Nurs*) \| 1223462 \| \| \|  \| 7. \| (“nursing care”) \| 62946 \| \| \|  \| 8. \| (MH “Nursing care”) \| 31449 \| \| \|  \| 9. \| (MH “Nurses”) \| 47955 \| \| \|  \| 10. \| S6 OR S7 OR S8 OR S9 \| 1221033 \| \| \|  \| 11. \| S5 AND S10 \| 1075 \| \| \|  \| 12. \| Peer Reviewed; English Language \| 1075 \| \| \|  \|  \|  \|  \| \| \| PuBMed 2024-11-25 \| \| \| \| \|  \| 1. \| (altruis*) \| 13233 \| \| \| --- \| --- \| --- \| --- \| --- \| \|  \| 2. \| (“altruistic behaviour*”) \| 169 \| \| \|  \| 3. \| (”altruistic behavior*”) \| 515 \| \| \|  \| 4. \| ("Altruism")"[Mesh] \| 8140 \| \| \|  \| 5. \| S1 OR S2 OR S3 OR S4 \| 13233 \| \| \|  \| 6. \| (Nurs*) \| 1213549 \| \| \|  \| 7. \| (“nursing care”) \| 61043 \| \| \|  \| 8. \| (“Nursing care”) [Mesh] \| 143726 \| \| \|  \| 9. \| (“Nurses”) [Mesh] \| 101762 \| \| \|  \| 10. \| S6 OR S7 OR S8 OR S9 \| 1214002 \| \| \|  \| 11. \| S5 AND S10 \| 1149 \| \| \|  \| 12. \| Peer Reviewed; English Language \| 1149 \| \| \|  \|  \|  \|  \| \| \| PsychInfo 2024-11-26 \| \| \| \| \|  \| 1. \| (altruis*) \| 12246 \| \| --- \| --- \| --- \| --- \| \|  \| 2. \| (“altruistic behaviour*”) \| 116 \| \|  \| 3. \| (”altruistic behavior*”) \| 1155 \| \|  \| 4. \| (DE "Altruism") \| 6515 \| \|  \| 5. \| S1 OR S2 OR S3 OR S4 \| 12246 \| \|  \| 6. \| (Nurs*) \| 221047 \| \|  \| 7. \| (“nursing care”) \| 7567 \| \|  \| 8. \| (DE “Nurses”) \| 37416 \| \|  \| 9. \| S6 OR S7 OR S8 \| 221047 \| \|  \| 11. \| S5 AND S9 \| 375 \| \|  \| 12. \| Peer Reviewed; English Language \| 375 \| \|  \|  \|  \|  \| \| \| \| \| \| \| \| \| | | | |
|  | | | |
