# Supplementary material for: Professional altruism in nursing care: A concept clarification study
Source: Int J Nurs Stud Adv. 2026 Mar 16;10:100522. doi: 10.1016/j.ijnsa.2026.100522 (PMC13053996; doi:10.1016/j.ijnsa.2026.100522)
Supplement: Supplementary file 2 [file mmc2.docx]

| **Supplementary Material File 2. Matrix of the included articles** | | | | | | |
| --- | --- | --- | --- | --- | --- | --- |
| **Article** | **Country** | **Aim** | **Design** | **Methods** | **Study population** | **Results** |
| Alavi et al. (2015)^1^ | Iran | To clarify paediatric nurses’ characteristics of caring self-efficacy | Qualitative inductive design | Semi-structured interviews  Content analysis (*Graneheim and Lundman*) | Volunteer nurses, nursing managers, and paediatric nurses (n=27) | The data analysis generated four main themes as attributes of a self-efficient paediatric nurse: (a) professional communications; (b) management of care; (c) altruism; and (d) proficiency. |
| Alavi et al. (2017) ^2^ | Iran | To introduce altruism as one of the main aspects of caring self‑efficacy in paediatric nurses | Qualitative inductive design | Semi‑structured interviews  Content analysis (*Graneheim and Lundman*) | Paediatric nurses, clinical directors, clinical nursing professors (n=27) | ‘Altruism’ is one of the main themes extracted from the analysis of the interviews in this study. This theme includes two main categories: ‘humanistic care’ and ‘caring attitude’. |
| Albuquerque et al. (2018)^3^ | Sweden | To understand what motivates and supports experienced humanitarian health workers to remain in the sector | Qualitative inductive design | Semi-structured interviews  Content analysis (*Graneheim and Lundman*) | Nurses whose last mission in humanitarian work had taken place within the last three years (n=10) | The findings indicate that the nurses’ motivations and how they thought of themselves as individuals and professionals changed over time. For initiation and continued engagement in humanitarian work, the participants were motivated by several personal and professional ambitions as well as altruistic principles of helping others. When starting their first humanitarian missions, the nurses felt vulnerable and had low self-esteem. However, through experiencing feelings of autonomy, competence, and relatedness during missions, they underwent a process of change and gradually adjusted to their new roles as humanitarian health workers. Reintegration in their home communities, while maintaining the new roles and skills from the missions, proved very challenging. Individually, they found their own ways of overcoming the lack of social support they experienced after missions in order to sustain their continuation in the sector. |
| Altun (2002)^4^ | Turkey | To make suggestions and to determine which values are of significance if and when nurses experience burnout | Quantitative descriptive study  Cross sectional design | A questionnaire was developed for the study.  The first part sought demographic data, the second part concerned seven professional values and attitudes, and the third part included questions about the definitions relating to Maslach’s Burnout Inventory.  Descriptive and comparative statistical analysis | Nurses working in Kocaeli district, Turkey (n=160) | The results show that nurses’ personal and professional values play an important role in the degree of burnout they experience. Equality, altruism, and aesthetics were ranked first by those experiencing high levels of emotional exhaustion, and freedom was a priority value for those with a low degree of emotional exhaustion. Freedom, altruism, and truth were ranked first by those with prominent feelings of personal accomplishment, while equality and aesthetics were priority values for those with a lesser feeling of personal accomplishment. All nurses, therefore, need to identify and clarify their own personal values, beliefs and assumptions about basic truths. |
| Atkinson (2015)^5^ | Kuwait | To illuminate the perception among Muslim nurses in Kuwait of the role of Islamic values on their nursing practice | Qualitative inductive design | Semi‑structured interviews  Thematic analysis (*Leininger & McFarland*) | (Muslim) nurses (n=18) | Seven themes emerged: (1) altruistic relationships as a core value; (2) all care as spiritual care; (3) a desire for greater understanding and respect as nurses and as Muslims; (4) professional kinship that transcends culture, religion, and nationality; (5) nursing ethics from divine ethics; (6) religious teachings promoting health; and (7) radical acceptance of God’s will, balanced with the hope of reward |
| Carter (2014)^6^ | United Kingdom | To understand the vocational and altruistic motivations of nurses through the application of Pierre Bourdieu’s concepts of ‘symbolic capital’, ‘field’ and ‘habitus’ through a long interview with nurse respondents | Qualitative inductive design | Interviews  Thematic analysis (*Bourdieu, Rhynas*) | Community nurses (n=12) | The nurse respondents had highly individual and at times contradictory views on their motivations to nurse, including their views on vocation and altruism in nursing careers. |
| Cross et al. (2020)^7^ | USA | To understand the experiences of nurses making the role transition from clinical nurse specialists (CNSs) (hospital based) into transitional care nurse (TCN) roles (community based) | Qualitative inductive design | Semi-structured interviews  Constant comparative method (*Glaser & Strauss*) | Transitional care nurses (n=5) | Seven major themes were identified: enhanced patient-centred care, collaboration among the other TCNs, transitioning from expert to novice, recommendations for navigating and negotiating systems, discomfort with the role transition, a level of altruism and autonomy, and recommendations for improving the TCN role. Minor themes supported the major themes. |
| De Cooman et al. (2008)^8^ | Belgium | To identify the features young nurses look for in their job (job motives) and the features they look for in work in general (work values) | Cross sectional design | Quantitative survey  Descriptive and comparative statistical analysis | Newly graduate nurses, response rate 30.1%  (n=344) | The results indicate that many of the traditional attractions are still important, with the recurrent issue of altruism. Furthermore, men are found to be more attracted by career opportunities, executive powers, and autonomy, while women tend to attach more importance to interpersonal characteristics. |
| Decoyna et al. (2018)^9^ | Australia | To enhance the understanding of nurses and physiotherapists' experience in mobilising postoperative orthopaedic patients with altered mental status | Qualitative inductive design | Semi-structured interviews  Thematic content analysis (*Burnard*) | Nurses in acute orthopaedic ward (n=3) | Four main categories emerged from the study: altruism, interprofessional specialist practice, patient dynamics, and challenges. Nurses’ and physiotherapists’ experiences have more similarities than differences under the four categories. |
| Dotson et al. (2014)^10^ | USA | To discover and quantify factors that influence RN job satisfaction, behavioural intentions (BIs) to leave a current job, and BIs to leave the nursing profession | Cross sectional design | A survey instrument was developed based on a review of the literature and focus group results  SEM (Structural equation modelling) analyse | Nurses, response rate 27.6% (n= 861) | The results confirm the importance of stress and salaries and underscore the impact of both value congruence and altruism. The evidence shows a correlation between altruistic desires and intentions to leave the profession. |
| Eder & Meyer (2023)^11^ | Germany | To expand the perspective of the Job Demand-Resources Model by including caregivers’ intraindividual resources and the coping construct of self-endangering as a mediator between personal resources and nurses’ emotional exhaustion | Cross sectional design | Questionnaire survey  Descriptive and comparative statistical analysis | Nurses in long-term care (n=416) | A highly altruistic job motivation leads to more self-endangering cognitions and behavioural tendencies. Mixed-model analysis and cross-sectional path analysis confirmed the mediation effects from altruism over self-endangering to exhaustion. |
| Fagermoen (1997)^12^ | Norway | What are the values underlying nurses' professional identity as expressed in what is experienced as meaningful in nurses' work?' | Mixed method design | *Part 1*  A dataset from a survey with open-ended questions  Descriptive and comparative analysis  *Part 2*  Interviews  Hermeneutic analyses | *Part 1*  Nurses (n=767)  *Part 2*  Nurses (n=6) | Content analysis of survey data revealed that the nurses held both other-oriented and self-oriented values (i.e. both moral and work values). Human dignity and altruism were the most prominent moral values, whereas the most significant work values were intellectual and personal stimulation. The interview data, analysed by means of hermeneutic and narrative analysis, revealed greater diversity in value expressions compared to the survey data. Altruism, the moral orientation of care, was the overall philosophy, and human dignity appeared as a core value. The nine additional values appeared to be linked to human dignity either by arising from it and/or being aimed at preserving this basic value. |
| Ghaljeh et al. (2024)^13^ | Iran | To investigate nurses’ understanding and experience of compassionate care when working in paediatric oncology departments | Qualitative inductive design | Semi-structured interviews  Content analysis (*Elo & Kyngäs*) | Nurses in paediatric oncology departments (n=32) | Nurses’ understanding of compassionate care for children with cancer was organised into two categories: humanistic compassionate care and compassionate end-of-life care. The humanistic compassionate care category consisted of three subcategories: (1) empathy, (2) altruism, and (3) respect for the cultural values and beliefs of the family. The compassionate end-of-life care category comprised two subcategories: (1) facilitating parents’ presence at the child’s bedside and (2) creating suitable conditions for accepting the death of a child. |
| Ghanbari-Afra et al. (2021)^14^ | Iran | To explore nurses' perceptions of CBC for COVID-19 patients  (*CBC – Compassion based care*) | Qualitative inductive design | Semi-structured interviews  Content analysis (*Elo & Kyngäs*) | Nurses in COVID-19 units (n=25) | In the care of COVID-19 patients, CBC consisted of three categories: prosocial behaviours, paying attention to the beliefs and values of patients, and concern for family members. The first category had three subcategories: empathy, altruism, and helping in critical situations. The second category included the subcategories of the spiritual approach to care and respect for cultural aspects. Finally, the third category, concern for family members, had one subcategory: the need to consider the patient’s family. |
| Hamooleh et al. (2013)^15^ | Iran | To explain nurses’ perception about ethics-based palliative care in cancer patients | Qualitative inductive design | Semi structured interview  Qualitative content analysis (*Graneheim & Lundman*) | Nurses in adult cancer ward (n=14) | In general, original categories in the ethics-based palliative care of cancer patients encompass the following: ‘human dignity’, ‘professional truthfulness’ and ‘altruism’. Human dignity has three subcategories consisting of ‘respecting patients’, ‘paying attention to patient values’, and ‘empathising’. Professional truthfulness has two subcategories: ‘truthful speech’ and ‘truthful action’. Lastly, altruism has three subcategories: ‘complete and multi-dimensional patient acceptance’, ‘supportive behaviour’, and ‘responsibility’. |
| Lazar (2010)^16^ | Israel | To examine the relationship between spirituality and hospital nurses’ work satisfaction and to determine the unique contributions of various specific aspects of spirituality to their work satisfaction | Quantitative inductive design | A questionnaire and a correlational design  Descriptive and comparative statistical analysis | Female nurses from a public hospital in a large city (n=120) | Correlation analysis indicated a positive relationship between the life coherency aspects of spirituality and spiritual values with job satisfaction. Hierarchical regression analysis indicated the particular importance of an idealistic spiritual orientation (positive contribution) and a transcendent spiritual orientation (negative contribution) in predicting nurses’ job satisfaction. |
| Pang et al. (2009)^17^ | China | To identify the essential professional values of Chinese nurses and their manifestations in the current health-care environment. | Qualitative inductive design | Focus groups and semi-structured individual interview  Thematic analysis (*Morse & Field*) | Senior clinical nurses, nurse administrators, or nurse educators in four tertiary hospitals and three university nursing schools in Beijing and Shanghai, China (n= 30) | Seven themes were identified: altruism, caring, trustworthiness, dignity, responsibility for the development of the profession, autonomy, and justice. On the whole, these values were in accordance with the codes of the International Council of Nurses and the Chinese Nursing Association. Additionally, culture and socioeconomic trends were found to influence nurses’ understanding and explanation of professional values. |
| Rantung (2025)^18^ | Indonesia | To explore how emergency nurses sustain themselves personally and professionally  (*aimed at exploring social processes*) | Qualitative inductive design | Semi-structured interviews  Constructivist grounded theory analysis (*Charmaz*) | Nurses from diverse backgrounds in the Indonesian emergency nursing context (n=29) | The study revealed three subcategories under the ‘Driving Forces’ category: ‘Inherent Inspiration’, ‘Extrinsic Motivations’, and ‘Professional Expectations’. These subcategories highlight the significant impact of both intrinsic and extrinsic motivations, along with professional expectations, on the commitment and sustainability of emergency nurses. |
| Shahoei et al. (2022)^19^ | Iran | To explore the experiences of nurses in providing care to patients with COVID-19. | Qualitative inductive design | Semi-structured interviews  Phenomenological method (*Colaizzi's*) | Nurses who had experience providing care to patients with COVID-19 (n=14) | The participants included four men and 10 women. The data analysis revealed four main themes (and 14 subthemes) related to the experience of the participants in providing care to patients with COVID-19. These themes included (a) fear (fear of being infected, fear of being a carrier, fear of the disease, and high mortality of patients), (b) compulsion (compulsion to care, and being under pressure), (c) distinct experience (need for self-care, working with personal protective equipment, and ambiguity in care/treatment), and (d) sacrifice (altruism, compassion, self-sacrifice, and being proud of yourself). |
| Slettmyr et al. (2022)^20^ | Sweden | To elucidate clinical expressions of ontological situational ethics through nurses’ willingness to work during a pandemic | Qualitative inductive design | Interviews designed with open questions and conversational in nature  Phenomenology and hermeneutics (*Lindseth & Norberg*) | Nurses who worked in intensive care unit taking care of patients with COVID-19 (n=20) | From a philosophical perspective, the nurses expressed sovereign life expressions of mercy and compassion, which arose spontaneously in response to seeing vulnerable fellow humans. They referenced ‘the nurse inside me’ and their choice of profession as motives for providing care. Ontological situational ethics in culture and norms were noted in the constructs of competence, responsibility, solidarity with colleagues and the organisation, and interest and learning were driving forces. Ethical demand was evident when nurses expressed ideas of meaningfulness in helping their fellow humans, but themes of ambiguity, exhaustion, and unwillingness were also present. |
| Slettmyr et al. (2019)^21^ | Sweden | To explore the meaning of altruism in nursing. | Qualitative inductive design | Socratic dialogues  Phenomenological hermeneutical method (*Lindseth & Norberg*) | Nurses from a Swedish acute care settings (n=13) | Altruism created a sense of ambivalence and ambiguity, described as a rise of sovereign expressions of life caused by ‘the other’s’ need but also an unwillingness to take unconditional responsibility for ‘the other’. |
| Vishnevsky et al. (2015)^22^ | USA | To examine whether oncology nurses experience personal growth and wisdom as a result of caring for patients | Qualitative inductive design | Interviews  Analysis guided by basic grounded theory principles (*Strauss and Corbin*) | Nurses from oncology floor (n=30) | The subthemes of personal growth were largely consistent with the documented domains of posttraumatic growth and included the appreciation of life, a new perspective on life, relating to others, spiritual/religious growth, and personal strength. Subthemes of wisdom were more varied, reflecting the diversity of this construct in the context of nursing. Benevolence arose as a unifying theme between personal growth and wisdom, with subthemes centring on altruistic attitudes and behaviour towards patients and the greater community. The findings suggest that nurses develop personal growth, wisdom, and benevolence as a result of the emotional connections formed with patients and the subsequent struggle to cope with their loss. This process accords well with findings in other populations experiencing trauma and adversity directly. |
| Zarea et al. (2013)^23^ | Iran | To explore the experiences of nurses, who provide such care for mentally ill people, within the context of | Qualitative inductive design | Interviews  Hermeneutic analysis (*Diekelmann*)  MAXQDA-10 software | Mental health nurses (n=10) | Four themes and five subthemes were identified: ‘being engaged with patients’ (subthemes: ‘struggle for monitor/control’, ‘safety/security concerns’, and ‘supporting physiological and emotional needs’), ‘being competent’, ‘altruistic care’, and ‘facing difficulties and challenges’ (subthemes: ‘socio-cultural’ and ‘organisational challenges’). The results provide valuable insights and a greater understanding of the professional experiences of psychiatric nurses in Iran and indicate the need for a stable and responsible organisational structure for those nurses who are expected to manage patient care in psychiatric wards. |
| Özsaban et al. (2024)^24^ | Turkey | To examine the relationship between the motivation, altruism and professional commitment levels of nurses during the COVID-19 pandemic | Cross sectional study | Questionnaire - Nurse Information Form, Nurse Job Motivation Scale, Altruism Scale and Nursing Professional Commitment Scale based on self-report  R version 2.15.3  Descriptive and comparative statistical analysis | Nurses in a university hospital (n=241) | The nurses included in this study had an average score of 66.56 (SD = 7.70) on the Nurses Job Motivation Scale, 70.24 (SD = 10.5) on the Altruism Scale, and 77.81 (SD = 12.56) on the Nursing Professional Commitment Scale. Statistically significant relationships were found between nurses’ motivation and altruism (p < 0.001). |

**References**

1. Alavi A, Bahrami M, Zargham-Boroujeni A, et al. Characteristics of caring self-efficacy in pediatric nurses: a qualitative study. *Journal for specialists in pediatric nursing : JSPN* 2015; 20: 157-164. DOI: 10.1111/jspn.12110.

2. Alavi A, Zargham-Boroujeni A, Yousefy A, et al. Altruism, the values dimension of caring self-efficacy concept in Iranian pediatric nurses. *Journal of education and health promotion* 2017; 6: 8. DOI: 10.4103/jehp.jehp_142_14.

3. Albuquerque S, Eriksson A and Alvesson HM. The rite of passage of becoming a humanitarian health worker: experiences of retention in Sweden. *Global health action* 2018; 11: 1417522. DOI: 10.1080/16549716.2017.1417522.

4. Altun I. Burnout and nurses' personal and professional values. *Nursing ethics* 2002; 9: 269-278. DOI: 10.1191/0969733002ne509oa.

5. Atkinson C. Islamic Values and Nursing Practice in Kuwait. *Journal of holistic nursing* 2015; 33: 195-204. DOI: 10.1177/0898010114564682.

6. Carter M. Vocation and altruism in nursing: the habits of practice. *Nursing ethics* 2014; 21: 695-706. DOI: 10.1177/0969733013516159.

7. Cross KL, Johnson P, Allard BL, et al. Clinical Nurse Specialists' Perceptions of Transitioning Into a Rural Community-Based Transitional Care Role. *Journal of nursing administration* 2020; 50: 456-461. DOI: 10.1097/NNA.0000000000000916.

8. De Cooman R, De Gieter S, Pepermans R, et al. Freshmen in nursing: job motives and work values of a new generation. *Journal of nursing management* 2008; 16: 56-64. DOI: 10.1111/j.1365-2934.2007.00800.x.

9. Decoyna JAA, McLiesh P and Salamon YM. Nurses and physiotherapists' experience in mobilising postoperative orthopaedic patients with altered mental status: A phenomenological study. *Int J Orthop Trauma Nurs* 2018; 29: 32-40. DOI: 10.1016/j.ijotn.2018.02.005.

10. Dotson MJ, Dave DS, Cazier JA, et al. An empirical analysis of nurse retention: what keeps RNs in nursing? *Journal of nursing administration* 2014; 44: 111-116. DOI: 10.1097/NNA.0000000000000034.

11. Eder LL and Meyer B. The role of self-endangering cognitions between long-term care nurses' altruistic job motives and exhaustion. *Front Health Serv* 2023; 3: 1100225. DOI: 10.3389/frhs.2023.1100225.

12. Fagermoen MS. Professional identity: values embedded in meaningful nursing practice. *Journal of advanced nursing* 1997; 25: 434-441. DOI: 10.1046/j.1365-2648.1997.1997025434.x.

13. Ghaljeh M, Mardani-Hamooleh M and Pezaro S. Nurses' perceptions of compassionate care in pediatric oncology: a qualitative interview study. *BMC health services research* 2024; 24: 1165. DOI: 10.1186/s12913-024-11661-1.

14. Ghanbari-Afra L, Salamat A, Hamidi H, et al. Compassion-based care for COVID-19 patients: a qualitative analysis of nurses' perceptions. *Journal of medical ethics and history of medicine* 2021; 14: 19. DOI: 10.18502/jmehm.v14i19.8179.

15. Hamooleh MM, Borimnejad L, Seyedfatemi N, et al. Perception of Iranian nurses regarding ethics-based palliative care in cancer patients. *Journal of medical ethics and history of medicine* 2013; 6.

16. Lazar A. Spirituality and job satisfaction among female Jewish Israeli hospital nurses. *Journal of advanced nursing* 2010; 66: 334-344. DOI: 10.1111/j.1365-2648.2009.05172.x.

17. Pang D, Senaratana W, Kunaviktikul W, et al. Nursing values in China: the expectations of registered nurses. *Nursing & health sciences* 2009; 11: 312-317. DOI: 10.1111/j.1442-2018.2009.00468.x.

18. Rantung GAJ. Uncovering the Driving Forces of Emergency Nurses' Retention: Findings From a Grounded Theory Study. *Journal of emergency nursing* 2025; 51: 220-228. DOI: 10.1016/j.jen.2024.10.003.

19. Shahoei R, Nemati SM and Valiee S. Exploring the Experience of Nurses in Providing Care to Patients With COVID-19: A Qualitative Study. *The journal of nursing research : JNR* 2022; 30: e217. DOI: 10.1097/jnr.0000000000000498.

20. Slettmyr A, Schandl A, Andermo S, et al. Spontaneous ethics in nurses' willingness to work during a pandemic. *Nursing ethics* 2022; 29: 1293-1303. DOI: 10.1177/09697330221085768.

21. Slettmyr A, Schandl A and Arman M. The ambiguity of altruism in nursing: A qualitative study. *Nursing ethics* 2019; 26: 368-377. DOI: 10.1177/0969733017709336.

22. Vishnevsky T, Quinlan MM, Kilmer RP, et al. “The Keepers of Stories”. *Journal of holistic nursing* 2015; 33: 326-344. DOI: 10.1177/0898010115574196.

23. Zarea K, Nikbakht-Nasrabadi A, Abbaszadeh A, et al. Psychiatric nursing as 'different' care: experience of Iranian mental health nurses in inpatient psychiatric wards. *Journal of psychiatric and mental health nursing* 2013; 20: 124-133. DOI: 10.1111/j.1365-2850.2012.01891.x.

24. Özsaban A, Turan N, Kıyak Y, et al. Nurses’ Motivation, Altruism and Professional Commitment Levels During the Pandemic: A Descriptive and Correlational Study. *Journal of Health and Nursing Management* 2024; 11: 313-322. DOI: 10.54304/SHYD.2024.43179.
