# Supplementary material for: Professional altruism in nursing care: A concept clarification study
Source: Int J Nurs Stud Adv. 2026 Mar 16;10:100522. doi: 10.1016/j.ijnsa.2026.100522 (PMC13053996; doi:10.1016/j.ijnsa.2026.100522)
Supplement: Supplementary file 3 [file mmc3.docx]

Supplementary Material File 3. Example of systematic analysis

| **Meaning units** | **Codes** | **Sub-category** | **Category** |
| --- | --- | --- | --- |
| ... altruistic & interpersonal is considered as the most important job motive (DeCooman et al. 2008)  Altruism and Idealism. Both of these dimensions demonstrated strong correlations with job satisfaction.(Lazar, 2010) | Altruism one important job motive  Strong correlation with job satisfaction | Contributes to job satisfaction | A motivational force |
| Altruism implied a feeling of “making a difference for the other person” and thereby creating a sense of meaningfulness in their work. This motivated them to further altruistic behavior. (Slettmyr et al., 2019)  All participants described feeling as if they were making a difference,(Cross et al., 2020) | Giving a sense of meaningfulness to work  Making a difference | Generates valued rewards |  |
| Driving forces play a crucial role in the sustainability of emergency nurses, emphasizing their importance in shaping policies, practice, and support systems within health care settings (Rantung, 2025).  These spontaneous altruistic actions saved the lives of many patients during the pandemic and need to be understood and supported.(Slettmyr et al., 2022) | A crucial driving force in nursing sustainability in health care (support it)  A contributing factor to many patients’ lives saved during the pandemic | Provides positive outcomes for nursing |  |
