# Supplementary material for: Professional altruism in nursing care: A concept clarification study
Source: Int J Nurs Stud Adv. 2026 Mar 16;10:100522. doi: 10.1016/j.ijnsa.2026.100522 (PMC13053996; doi:10.1016/j.ijnsa.2026.100522)
Supplement: Supplementary file 4 [file mmc4.docx]

**Supplementary Material File 4. References included in the categories in step two**

| **Reference** | **A willingness to act for others** | **A moral orientation** | **A motivational force** | **An unwavering professional expectation** | **A valued, yet challenged and sometimes rejected phenomenon** |
| --- | --- | --- | --- | --- | --- |
| Alavi et al. (2015) | X |  | X | X |  |
| Alavi et al. (2017) | X |  |  |  |  |
| Albuquerque et al. (2018) |  |  | X |  |  |
| Altun (2002) | X |  | X | X | X |
| Atkinson (2015) |  | X |  | X |  |
| Carter (2014) |  |  | X | X | X |
| Cross et al. (2020) |  | X | X |  |  |
| De Cooman et al. (2008) |  |  | X |  |  |
| Decoyna et al. (2018) | X | X |  |  |  |
| Dotson et al. (2014) |  |  | X | X | X |
| Eder & Meyer (2023) |  |  |  |  | X |
| Fagermoen (1997) | X | X | X |  |  |
| Ghaljeh et al. (2024) | X |  | X | X |  |
| Ghanbari-Afra et al. (2021) | X |  |  | X |  |
| Hamooleh et al. (2013) | X | X |  | X |  |
| Lazar (2010) |  |  | X | X |  |
| Pang et al. (2009) | X |  | X |  |  |
| Rantung (2025) |  | X | X |  |  |
| Shahoei et al. (2022) | X | X | X |  |  |
| Slettmyr et al. (2022) | X | X | X |  |  |
| Slettmyr et al. (2019) | X |  | X | X | X |
| Vishnevsky et al. (2015) | X | X | X | X |  |
| Zarea et al. (2013) |  | X | X |  |  |
| Özsaban et al. (2024) |  | X | X | X |  |
